# Wireless Capsule Endoscopy for Diagnosis and Management of Post-Operative Recurrence of Crohn’s Disease

**DOI:** 10.3390/life11070602

**Published:** 2021-06-23

**Authors:** Adil Mir, Vu Q. Nguyen, Youssef Soliman, Dario Sorrentino

**Affiliations:** 1 IBD Center, Division of Gastroenterology, Virginia Tech Carilion School of Medicine, Roanoke, VA 24016, USA; asmir@carilionclinic.org (A.M.); vqnguyen@carilionclinic.org (V.Q.N.); yysoliman@carilionclinic.org (Y.S.); 2 Department of Clinical and Experimental Medical Sciences, University of Udine School of Medicine, 33100 Udine, Italy

**Keywords:** Crohn’s disease, capsule endoscopy, ileo-colonoscopy, post-operative Crohn’s disease

## Abstract

Despite aggressive medical therapy, many patients with Crohn’s disease require surgical intervention over time. After surgical resection, disease recurrence is common. Ileo-colonoscopy and the Rutgeerts score are commonly used for diagnosis and monitoring of post-operative endoscopic recurrence. The latter is the precursor of clinical recurrence and therefore it impacts prognosis and patient management. However, due to the limited length of bowel assessed by ileo-colonoscopy, this procedure can miss out-of-reach, more proximal lesions in the small bowel. This limitation introduces an important uncertainty when evaluating post-operative relapse by ileo-colonoscopy. In addition, the Rutgeerts score ‘per se’ bears a number of ambiguities. Here we will discuss the pros and cons of ileo-colonoscopy and other imaging studies including wireless capsule endoscopy to diagnose and manage post-operative recurrence of Crohn’s disease. A number of studies provide evidence that wireless capsule endoscopy is a potentially more accurate as well as less invasive and less costly alternative to conventional techniques including ileo-colonoscopy.

## 1. Introduction

Crohn’s disease (CD) is a chronic inflammatory disease that most commonly affects the terminal ileum and proximal colon but can affect any segment of the gut from the mouth to the anus [1,2]. It has been estimated that CD may affect the small bowel in up to 80% of cases and it can be limited to the small intestine in up to 30% of cases [3]. Diagnosis and management often involve multiple invasive and non-invasive tests [2,3,4,5]. Despite aggressive medical treatment, a large number of CD patients need surgical intervention during the course of the disease, due to various complications including strictures, obstruction, perforation, fistulae, abscess formation, or failure of medical treatment [2,3,4]. Although data show that the rate of surgery in patients with CD has declined over the past few decades [6], surgery is still frequently performed in CD patients and in addition post-operative recurrence (POR) is common [7,8,9,10]. Ileo-colonoscopy [IC] at 6 to 12 months following surgery is widely used for the assessment of endoscopic recurrence [7]. However, the length of bowel that can be endoscopically examined by IC is limited to the colon and terminal ileum. Hence, a number of imaging techniques have been used for this purpose. First approved in 2001 by the Food and Drug Administration (FDA), wireless capsule endoscopy (WCE) has also been used to assess portions of bowel that are not otherwise accessible by upper and lower gastrointestinal endoscopy in a number of clinical conditions [11]. Here we will review the frequency and risk factors of POR in CD, the potential issues with the Rutgeerts endoscopic score and we will then focus on the use of WCE in the diagnosis and management of small bowel inflammation—especially POR—compared to IC and other imaging studies.

## 2. POR and Risk Factors

Even though complete clinical remission may be achieved after surgical resection, most patients eventually relapse, often needing medical or repeat surgical treatment.

In patients bound to develop relapse, D’Haens et al. have shown that focal infiltration of inflammatory cells including polymorphonuclear cells, mononuclear cells and eosinophils into the lamina propria is induced by contact with intestinal fluids within a period of 8 days [12]. Endoscopic post-operative recurrence (EPOR) is defined by visible macroscopic inflammation. EPOR rates vary among studies with ~60% of patients being affected at 1 year and up to 90% at 10 years [13]. Of the patients with EPOR, the majority will develop clinical POR (CPOR), defined by worsening clinical symptoms—usually diarrhea, and/or pain after surgery—over time, with rates approaching 75% by 10 years [9,10,14,15,16]. Hence, although inflammation does not always lead to clinical symptoms, EPOR is the obvious precursor of CPOR in all cases and timely diagnosis and management is required. Finally, surgical POR (SPOR) refers to the need for a repeat surgical procedure. About 25% of patients at 5 years and 35% of patients at 10 years might require repeat operative interventions [8].

A number of risk factors have been associated with POR in CD. These include smoking, aggressive and penetrating disease, previous surgical resection for CD, perianal disease and myenteric plexitis [16,17,18]. In addition, a shorter disease duration prior to surgery and proximal disease involving the duodenum or jejunum have been associated with an increased risk of recurrence [19,20]. Smoking seems to be the strongest and the only modifiable risk factor for POR in CD [17].

## 3. Diagnosis of POR in CD

We and others have shown that early diagnosis and treatment are associated with better clinical outcomes in CD [21]. In the case of CPOR it is essential to accurately diagnose EPOR (the precursor of CPOR) and define the location, extent and severity of mucosal inflammation. In clinical practice IC at 6–12 months from the operative intervention is widely used for post-operative diagnosis and monitoring of EPOR in CD [7]. An endoscopic scoring system proposed by Rutgeerts et al. is also widely used and is based on lesions of the neo-terminal ileum identified by IC [9,10,22]. The main features of the Rutgeerts score are illustrated in Table 1.

The risk of CPOR with a score of i0–i1 is reported to be <10% in 10 years; with a score i2 of 40% in 5 years, and with a score i3–i4 of 50–100% in 5 years [9,10].

The remarkable, landmark studies by Rutgeerts et al. were conducted almost 4 decades ago and have been the basis of most recent clinical trials in POR of CD. They have the added value of having been conducted essentially in the absence of major confounding factors—such as the use of effective medications to prevent POR, which were not available at that time. However, this score has never been formally validated and it is not officially recognized as a tool predictive of CPOR. In addition, there are a number of other issues with this score. First, in the original studies, small bowel lesions were evaluated only when visible lesions extended beyond the reach of the endoscope and only by barium meal follow-through (an inaccurate imaging technique especially for superficial mucosal lesions) [9,10]. Obviously, at that time, more accurate imaging modalities were not available. Hence, it is possible that isolated lesions in the small bowel were not taken into account during both the initial as well as the pre-operative and the post-operative disease staging. As a consequence, it is possible that some of the patients with the lowest scores had small bowel disease or that some of the patients with the highest scores were symptomatic due to the presence of additional, undetected small bowel disease.

Second, the study sample size was quite small, especially for the groups that had a score of i1, i2 and i3 (n = 11, 15 and 16 respectively) [10]. Third, it is unclear how the individual endoscopic scores measured at index IC could really predict CPOR. In fact, even though the authors show that a sizeable proportion of patients with an endoscopic score of i0 at 1 year evolved into scores i3 and i4 at year 3, the rate of CPOR in the long term was still reported to be negligible for patients with a score of i0 [10]. Likewise, approximately 40% of patients with score i2 were reported to convert to score i4 at 4 years yet only 40% of these i2-patients were reported to develop CPOR at the end of follow up (8 years) [9,10]. Fourth, the practice of separating endoscopic remission (scores i0 and i1) from endoscopic relapse (scores i2–i4) based on the presence of a single aphthous ulcer appears to be highly precarious to predict CPOR and planning the management strategy. Indeed, due to these uncertainties, the Rutgeerts score is now rarely, if ever, recommended as a single endpoint in trials probing medications to prevent CPOR.

## 4. WCE and Other Tests to Diagnose EPOR

As discussed above, CD can affect any segment of the entire digestive tract [1,2,3,4,23] including large portions that are not reached by IC. Obviously, missing more proximal lesions can lead to inaccurate staging and treatment of the disease process before surgery. Such lesions would also be missed by IC after surgery. Yet, IC is widely considered accurate in ≥90% of cases to detect EPOR. WCE offers a potential diagnostic advantage compared to IC since it can visualize the entire length of the bowel. Recent literature has offered insight into the use of WCE in the detection of small bowel inflammation and EPOR in CD. These studies are summarized in Table 2 and reported in detail below.

In a multicenter prospective study, Bruining et al. compared the sensitivity and specificity of panenteric WCE (PillCam Crohn’s system—Medtronic) with magnetic resonance enterography (MRE) and/or IC [24] in patients with established CD. 158 subjects from 3 different countries (USA, Austria, and Israel) were enrolled in the study out of whom 99 were included in the analysis. Test results were interpreted by blinded central readers using standardized scoring systems. Overall sensitivity for active enteric inflammation (WCE vs. MRE and/or IC) was 94% vs. 100% (*p* = 0.125) and specificity was 74% vs. 22% (*p* = 0.001). However, sensitivity of WCE was higher than MRE for proximal bowel inflammation (97% vs. 71%, *p* = 0.021), and similar to MRE and/or IC for the terminal ileum and colon (*p* = 0.500–0.625) [24]. WCE had a higher positive predictive value (and lower negative predictive value) than MRE and/or IC [24]. A total of 7 adverse events (AE) were reported but only 3 events were thought to be related to the WCE including abdominal pain, partial bowel obstruction and perforation, secondary to retained capsule [24]. Notably, only patients with suspected strictures underwent patency capsule before WCE. In our own practice, we routinely exclude potential issues in all patients before WCE by patency capsule.

**Figure 1:**
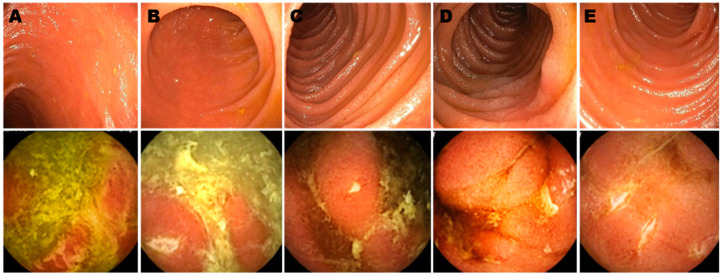
Ileo-colonoscopy (upper panels) and wireless capsule endoscopy (lower panels) images in Crohn’s disease patients after surgery. Even though all these patients were assumed to be in endoscopic remission by IC and the Rutgeerts score (which was i0 in (**B**–**D**) and i1 in (**A**,**E**)), significant inflammation—proximal to the reach of the colonoscope—was demonstrated by wireless capsule endoscopy, which was performed within 8 weeks from/to ileo-colonoscopy (Reprinted with permission from reference [25], Oxford University Press 2018.).

Our group has recently conducted a retrospective study involving a total of 43 patients who underwent WCE due to symptoms that were unexplained by other standard diagnostic modalities including IC and imaging (CT enterography [CTE] and MRE) [25]. We enrolled a total of 43 patients, 25 of whom had had surgery. In patients who never had surgery, imaging was negative with a positive WCE in 8/15 (53%) of cases. Colonoscopy was insufficient for disease staging in 10/20 (50%) of the cases. CRP and fecal inflammatory markers were normal with a positive WCE in 35% and 28% of cases, respectively. WCE findings changed the management in 6/20 (30%) cases with 83% showing clinical/biochemical improvement after up to 15 months of follow-up. In post-operative patients (25 out of 43) imaging was negative and WCE was positive in 75% of cases [25]. Colonoscopy was inaccurate for disease staging in 59% of cases (Figure 1). In patients with positive WCE findings, CRP and fecal markers of inflammation were within normal limits in 42% and 32% of cases, respectively [23]. Incorporation of WCE as a diagnostic modality changed management in 52% of the cases, which subsequently translated into clinical and biochemical improvement in 83% of them at follow up (up to 18 months) [25]. No adverse events were reported with WCE in this study.

Hausmann et al. conducted a prospective multicenter pilot study comparing pan-intestinal WCE with IC for the detection of POR in CD [26]. Out of the 16 patients who successfully underwent WCE, 3 patients had active disease at 4–8 weeks, with one patient having significant additional inflammation in the proximal bowel. Subsequently, at 4–8 months interval, 14 patients (out of whom 12 could be successfully followed) underwent WCE [26]. Findings included no inflammation in 4 (33%), mild disease activity (RS i1) in 2 (17%), moderate disease activity in 1 (8%) and severe disease activity in 5 (41%)—hence, 6 patients had active (moderate/severe) disease by WCE (including 1 patient with inflammation exclusively in the proximal small bowel) [26]. IC was performed in 15 of these patients at 4–8 months. Findings included no inflammation in 6 (40%), mild disease activity (RS i1) in 4 (26%), moderate disease activity in 2 (13%) and severe disease activity in 3 (20%)—hence, 5 patients out of 15 (33%) had active (moderate/severe) disease by IC [26]. Therefore, all the cases positive for active disease at IC were also detected by WCE. The patient with active disease in the proximal small bowel (only detected by WCE) had no significant inflammation of the neo-terminal ileum on IC [26]. The detection of bowel inflammation by WCE had a significant impact on the patients’ clinical management including initiation of adalimumab in 2 patients and azathioprine in 1 patient at 4–8 weeks [26]. Similarly, at the second surveillance interval (4–8 months), diagnostic findings changed the patients’ management including initiation of adalimumab in 4 patients and increase in the dose of azathioprine in one patient [26]. No adverse events were noted with the use of WCE [26].

A systematic review and meta-analysis by Yung et al. included a total of 14 studies which compared WCE, US and MRE with IC (using the RS) for the detection of EPOR [5]. Of these, 5 studies (including a total of 76 patients) compared WCE with IC; 3 studies (also including 76 patients) compared MRE with IC and 6 studies used US. The pooled sensitivity for WCE was 100%, pooled specificity was 69%, pooled diagnostic odds ratio (DOR) was 30.8 [5]. The area under the curve (AUC) was 0.94, representing high accuracy of WCE for detecting POR. In comparison, MRE had a pooled sensitivity of 97.3% and a pooled specificity of 83.7%, with pooled DOR of 129.5 and AUC of 0.98 (note that the included studies had low heterogeneity). US pooled sensitivity was 89% (95% CI, 85–92%), specificity 86% (95% CI, 78–93%), DOR 42.3 (95% CI, 18.6–96.0), and AUC 0.93 [5].

In a prospective study by Bourreille et al., IC and WCE were performed at 6 months after surgery in 32 patients [27]. Seven of these patients were on aminosalicylates and 3 were on immunosuppressants [27]. EPOR (which was defined as RS ≥ 1) was noted to occur in a total of 21 patients (68%) in the distal ileum. IC was able to detect EPOR in 19/21 patients (i1, i2 and i3 in 7, 6 and 6 patients, respectively). 10/21 patients had concurrent jejunal lesions (detected by WCE) alongside ileal recurrence [27]. In this study the sensitivity of WCE in detecting recurrence in the neoterminal ileum was inferior to that of IC. In contrast, WCE detected lesions outside of the scope of IC in more than two-thirds of patients with excellent interobserver agreement (kappa 0.9) for all lesions with the exception of ulceration (kappa = 0.7) [27]. It should be noted that this study was published 15 years ago and considerable WCE technical advancements have been made since.

Kusaka et al. performed WCE in 25 patients within three months after surgery for CD [28]. 21/25 patients (84%) had endoscopic activity (based on the Lewis score as per Gralnek et al. [29]). In addition, 5/25 patients developed CPOR over time. No adverse events related to WCE were noted. The cumulative CPOR rate was significantly higher in patients with the highest third tertile score (*p* = 0.046) [28]. Residual lesions after surgery, especially in the distal small intestine were associated with higher rates of CPOR, thus reinforcing the concept that those lesions are clinically significant and that early detection of EPOR is crucial to prevent CPOR [28].

In a retrospective cohort study by Han et al. [30], a group of 37 patients underwent IC with WCE (group 1) after 1 year of surgery and another group of 46 patients (group 2) underwent IC only. Patients who demonstrated evidence of EPOR (detected by IC and/or WCE), received pharmacologic therapy and had disease activity re-evaluated after 1 year [30]. In group 1, WCE was able to detect recurrence in all cases in which IC identified the recurrence. Importantly, WCE was able to detect EPOR (missed by IC) in 11 additional patients [30]. Furthermore, endoscopic remission diagnosed by IC was confirmed by WCE in 13 patients (in a total of 24 patients without recurrence). In comparison, in group 2, 31/46 patients were in clinical remission as diagnosed by IC. Out of these 31 patients 9 developed both EPOR and CPOR at 1 year follow up. The total CPOR rate at 1 year follow-up in group 1 was 2.7% (1/37) compared to 21.7% (10/46) in group 2 [30]. The authors concluded that if endoscopic remission was confirmed by WCE, the patients could remain free of pharmacologic prophylaxis, and conversely, if WCE detected recurrence missed by IC, starting pharmacologic therapy would be indicated [30].

## 5. Discussion

Post-operative monitoring, diagnosis, staging and treatment can be challenging in CD. Various modalities of small bowel imaging have been used over the years including small bowel follow through (SBFT), small bowel enteroclysis (SBE), small intestine contrast ultrasonography (SICUS), CTE and MRE [1,2,3,4,5,31,32]. None of these modalities allow for direct visualization of the bowel mucosa. SBE is more invasive and associated with higher radiation exposure compared to SBFT along with the added inconvenience of the naso-enteric tube placement. Both SBFT and SBE only provide limited information regarding bowel wall inflammation [31]. SICUS does not involve ionizing radiations, and it is a relatively well tolerated, non-invasive modality for small bowel assessment in CD. However, besides being highly operator dependent, bowel exam by SICUS may be hampered by bowel air/volume and it may be difficult to assess the proximal jejunum and ileum due to overlying bowel loops and their deep location [31]. CTE is a well-tolerated and less time-consuming diagnostic option. However, exposure to harmful ionizing radiation is a limitation of CTE [31]. Furthermore, superficial erosions/ulcers might not be easily visualized with CTE. MRE has been widely used for CD staging and it is considered the gold standard to assess small bowel disease in CD [33]. However, MRE—like CTE—is accurate mostly for transmural inflammation and early lesions limited to the mucosal surface may be missed by this imaging technique [25,34].

Due to its limited reach, a proportion of clinically significant inflammatory lesions can be missed if IC is used as the sole modality to check POR in CD [15,25,35]. In addition, IC is an invasive procedure and requires anesthesia/sedation and close monitoring during sedation. Finally, IC requires patients to miss a number of workdays due to preparation, visits, testing and recovery. In general, patients are likely to prefer a single test that can look at the entire GI tract mucosa [24]. As a consequence, costs and patient comfort become significant issues. In a recent patient satisfaction questionnaire administered after the procedure, 54% of the patients preferred WCE and 36% preferred IC [24]. Traditionally, WCE has been used to visualize the small bowel only. However, the recently developed Crohn’s capsule can be used to survey both the small and large bowel at once [36]. The use of dual cameras in some of these devices is also likely to increase their diagnostic yield.

A major drawback of WCE is the inability to obtain tissue samples from the abnormal mucosa for histopathological diagnosis. However, such need is less compelling when evaluating a patient for POR rather than for the initial diagnosis. In addition, WCE should not be used in stricturing or fistulizing CD due to the risk of capsule retention and possible partial or complete bowel obstruction, perforation or need for surgery [11]. However, the use of a patency capsule prior to the actual WCE deployment usually provides adequate assessment of the luminal patency and presence of strictures [37] and it is strongly recommended for the initial diagnosis and staging [11]. By definition, such limitation does not apply after surgery if the strictured area has been successfully removed.

As shown above, the diagnostic sensitivity of IC (and by inference the prognostic value of the Rutgeerts score) might at times be insufficient. Yet, major clinical trials testing medications to prevent POR have focused on IC findings and the RS as well as the presence of symptoms. In these studies, a negative IC has been equated to lack of relapse even when symptoms are present. That is because in the absence of endoscopic findings at IC symptoms are often attributed to consequences of surgery (for example to short bowel, post-operative inflammation at site of resection, bacterial overgrowth, adhesions, bile salt diarrhea). Clearly, the presence of undetected small bowel inflammation could change the interpretation of the results of these studies [28,38]. In addition, it is believed that EPOR only occurs at the neo-terminal ileum after surgery [12]. However, the presence of additional small bowel lesions after surgery would question such a principle and potentially impact therapeutic interventions [39]. Currently, WCE in post-operative CD patients is recommend by some authors only in the presence of unexplained clinical symptoms or biochemical marker elevations [40]. However, this strategy could miss small bowel lesions in clinically asymptomatic patients. Lesions which could evolve if left untreated. Well designed, prospective studies [15] should be conducted to answer this question and possibly to verify the results of many previous trials.

## 6. Conclusions

In conclusion, the use of WCE to diagnose EPOR after surgery might increase the diagnostic accuracy of the current tests including IC and the Rutgeerts score and might greatly impact therapeutic decisions. Development of validated scores focused on lesions detected by WCE could help predict the potential risk of future CPOR. Current trials probing new medications to prevent CPOR should take into consideration that negative results at IC—with or without clinical symptoms—might not be sufficient to declare the patient in endoscopic remission.

## Figures and Tables

**Table 1:** Rutgeerts scoring system for assessment of EPOR in CD.

Rutgeerts Score		Endoscopic Findings at IC
Grade i0	Endoscopic Post-operative Remission	Normal mucosa
Grade i1		<5 Aphthous ulcers
Grade i2	Endoscopic Post-operative Recurrence (EPOR)	>5 Aphthous ulcers with normal intervening mucosa or large lesions confined to the anastomosis
Grade i3		Diffusely inflamed mucosa with aphthous ileitis
Grade i4		Diffuse inflammation, large ulcers/nodules/narrowing

**Table 2:** WCE for detection of small bowel inflammation in CD.

Study	Sample Size	Study Design	Comparisons	Results	Comments
Bruining et al. [24]	99	Multicenter prospective cohort	WCE * vs. MRE ^	For proximal bowel inflammation, sensitivity of WCE and MRE were 97% and 71%. For inflammation in terminal ileum and colon, sensitivity of WCE was similar to MRE and/or IC	3 adverse events were reported with WCE. Only patients with suspected strictures at MRE underwent patency capsule.
Sorrentino et al. [25]	43	Retrospective cohort	WCE vs. IC ^±^ and/or MRE/CTE ^∞^	WCE detected inflammation undetected by IC and imaging in 59% and 75% of patients, respectively.	WCE changed management in 52% of the cases resulting in clinical and biochemical improvement in 83% of them at follow up (up to 18 months)
Hausmann et al. [26]	16	Multicenter prospective cohort	WCE vs. IC	WCE detected inflammatory lesions early at 4–8 weeks after surgery. WCE detected 1 additional patient (out of 6) with inflammation compared to IC at 4–8 months after surgery.	WCE use changed management in 3 patients at 4–8 weeks and in 1 patient at 4–8 months.
Yung et al. [5]	5 studies including 76 patients	Systematic review and meta-analysis	WCE vs. IC	Pooled sensitivity for WCE was 100%, pooled specificity was 69%.	The definition of recurrence varied in different studies. The included studies were cross sectional.
Bourreille et al. [27]	32	Prospective cohort	WCE vs. IC	Sensitivity and specificity for WCE for post-operative recurrence at neo-terminal ileum were 62–76% and 90–100%. For IC they were 90% and 100%.	In 2/3 of patients, WCE detected inflammatory lesions in the small bowel proximal to the reach of the colonoscope.
Kusaka et al. [28]	25	Prospective cohort	WCE only	21/25 patients had EPOR ^β^ within 3 months after surgery.	The severity of inflammatory lesions in the distal small intestine was associated with CPOR ^∑^.
Han et al. [30]	37	Retrospective cohort	IC + WCE vs. IC only	WCE detected EPOR undetected by IC in 11 patients. Total CPOR was 2.7% (IC+WCE group) vs. 21.7% (IC only) at 1 year follow up.	The authors concluded that if recurrence was detected by WCE, starting pharmacologic therapy would result in lower risk of CPOR.

* WCE: wireless capsule endoscopy; ^ MRE: magnetic resonance enterography; ^±^ IC: ileo-colonoscopy; ^∞^ CTE: computed tomographic enterography; ^β^ EPOR: endoscopic post-operative recurrence; ^∑^ CPOR: clinical post-operative recurrence.

## Data Availability

Not applicable.
